# Hybrid Hosts Based on Sodium Alginate and Porous Clay Heterostructures for Drug Encapsulation

**DOI:** 10.3390/polym13162803

**Published:** 2021-08-20

**Authors:** Anda Ionelia Voicu (Mihai), Sorina Alexandra Gȃrea, Eugeniu Vasile, Adi Ghebaur, Horia Iovu

**Affiliations:** 1 Faculty of Applied Chemistry and Materials Science, University Politehnica of Bucharest, 1-7 Gh. Polizu Street, 011061 Bucharest, Romania; anda.voicu89@gmail.com (A.I.V.); adi.ghebaur@upb.ro (A.G.); horia.iovu@upb.ro (H.I.); 2 Advanced Polymer Materials Group, University Politehnica of Bucharest, 1-7 Gh. Polizu Street, 011061 Bucharest, Romania; 3 Department of Science and Engineering of Oxide Materials and Nanomaterials, Faculty of Applied Chemistry and Material Science, University Politehnica of Bucharest, 1-7 Polizu, 011061 Bucharest, Romania; eugeniuvasile@yahoo.com; 4 Academy of Romanian Scientists, 54 Splaiul Independentei, 050094 Bucharest, Romania

**Keywords:** hybrids, porous clay heterostructures, sodium alginate, drug, 5-Fluorouracil

## Abstract

In this study, some hybrid materials based on sodium alginate (NaAlg) and porous clay heterostructures (PCHs) were investigated as new hosts for 5-Fluorouracil (5-FU) encapsulation. The hybrid hosts were prepared by ionotropic gelation technique using different concentrations of PCHs (1, 3, and 10 wt%) in order to identify the optimal parameters for encapsulation and drug release. The obtained hybrid materials were characterized using FTIR Spectrometry, thermogravimetric analysis (TGA), scanning electron microscopy (SEM), and UV-Vis spectrometry to investigate the interactions of the raw materials involved in the preparation of hybrid hosts, the influence of PCHs concentrations on drug encapsulation efficiency and drug release profile. All the results show that the synthesized hybrid materials were able to load a high amount of 5-FU, the encapsulation efficiency and the release profile being influenced by the concentrations of PCHs.

## 1. Introduction

Cancer is a leading cause of morbidity and death worldwide, which is characterized by the growth of abnormal cells that multiply uncontrollably and have the capacity to destroy healthy cells [1,2]. Nanotechnology is the most important technology used to identify cancerous cells in the initial stage. Another important aspect of cancer therapy is the development of new materials able to minimize the side effects of therapeutic drugs and also to enhance the efficiency of cancer immunotherapy [3,4].

Fluorouracil (5-fluoro-2,4-pyrimidinedione, 5-FU) is a pyrimidine analog drug widely used in chemotherapy. This type of drug shows an important activity concerning solid tumors encountered in breast, lung, colon, brain tumor, pancreatic, liver, and stomach cancer [5,6]. The 5-FU drug exhibits significant disadvantages such as high toxicity, low light stability, short half-life, and low drug selectivity towards tumors. These disadvantages can be overcome by the development of controlled drug-release systems and through loading the 5-FU drug in inorganic clay and/or biopolymeric systems. Usually, 5-FU is available as an intravenous formulation. This type of administration of 5-FU is associated with different side effects such as psychological stress, hypertrophy, or atrophy, which cause damage in healthy tissues. Moreover, intravenous administration of the drug has been shown to cause severe gastrointestinal, hematological, cardiac, dermatological, and neuronal effects [6,7,8]. The encapsulation of 5-FU into the host system may reduce the side effects and makes possible its oral administration.

Natural polymers (polysaccharides, proteins, peptides, polyesters) are materials extensively used in drug delivery systems due to favorable properties such as good biocompatibility and biodegradability, lower toxicity, easy accessibility, the ability to form gels, biological activities, low immunogenicity, low cost, and targeting capacity [9,10]. The most widely used natural polymers in drug delivery system are polysaccharides because these materials exhibit an excellent ability to encapsulate various drug molecules and a good capacity to achieve a controlled drug release profile. Polysaccharides can also minimize the side-effects of drugs and enhance the pharmacokinetic profile [11]. They can also improve the stability of drug molecules, decrease the drugs premature degradation and also enhance intracellular penetration [12].

Sodium alginate (NaAlg) is an anionic heteropolysaccharide with a linear structure derived from brown seaweed, The structure of sodium alginate consists from (1-4) linked β-D-mannuronic acid and αL-guluronic acid monomers. NaAlg is a pure natural biodegradable polymer characterized by attractive properties such as biocompatibility, hydrophilicity, relatively low expense, high swelling capacity, and cell attachment ability. It is also a non-toxic material to the human body [13,14,15,16,17,18,19,20,21,22]. Unfortunately, NaAlg presents disadvantages such as a strong hydrophilic character and loss of structural integrity. Additionally, being a pH sensitive polymer in an acidic environment, it tends to shrink, leading to a reduction of the bead size. This decrease is another drawback because the release of the encapsulated drug in NaAlg is lower in gastric fluid. Moreover, the dissolution of the polymer in basic conditions is another limitation of NaAlg [23]. To overcome the disadvantages of the polymer (Na-Alg) and drug (5-FU) limits, new strategies have been developed to obtained materials with special properties. These involve intercalation and reinforcement of sodium alginate with other polymers (gelatin, polyvinyl alcohol, chitosan [24], carrageenan [25], and pectin [26]) or dispersion of micro- and nano-structures (magnesium aluminum silicate [27] montmorillonite (MMT), [28,29], halloysite (HNT) [30,31], mesoporous silica [32], layered double hydroxide (LDH), rectorite, hydroxyapatite [33], graphene oxide (GO) [34,35], and carbon nanotubes (CNT) [36]) within the polymer matrix.

The biomedical applications of NaAlg include drug delivery vehicles for active pharmaceutical ingredients [37]; excipients (e.g., binders) for preparation of tablets or capsules; scaffolds for cell culture and tissue engineering; and model extracellular matrices for biological studies, drug delivery, and magnetic resonance imaging [19,38].

Recently, hybrid nanoparticles based on NaAlg and organic/inorganic compounds were widely used in drug delivery systems because they improve drug release profile, compatibility, and swelling properties [39,40,41]. Good biocompatibility, tunable mechanical properties, and easy degradation are extraordinary and versatile properties for pharmaceutical purposes. Additionally, these systems have been synthesized to sustain a suitable amount of the drug without causing toxicity or leading to its effects being below the minimum effective level [42].

First, their small size and large surface lead to a higher absorption capacity of the drug and better drug controlled release compared with large carrier [43]. Second, the nanoparticle surface can be modified by chemical functionalization with various molecules in order to increase the drug absorption and release. Third, the introduction of an inorganic guest such as MMT within the polymer matrix could enhance the stability of the alginate and preserves its original biological function. The physical properties of the alginate beads could be improved by dispersing the magnesium aluminum silicate (MAS) due to the interaction of Na-Alg with silanol groups of MAS [44]. Drug delivery systems (DDS) based on NaAlg- nano-microparticles, composite microparticles, and biodegradable hydrogels are some examples that offer special features such as controlled drug release, improved pH sensitivity, and improved swelling capacity [32,36,45,46,47].

Porous clay heterostructures (PCHs) are inorganic materials, prepared for the first time in 1995 by Galarneau et al. [48]. The PCHs synthesis is based on three main steps: (1) intercalation of surfactant (quaternary alkylammonium cations) and co-surfactant (neutral amine) into layered inorganic clay, (2) hydrolysis and condensation of silica precursor (tetraethyl orthosilicate) in the clay gallery, and (3) thermal treatment or solvent extraction in order to remove the organic compounds [49,50,51,52,53]. PCHs are innovative materials with versatile properties such as: tunable textural properties (high surface area, microporosity, and mesoporosity) [54,55,56,57,58], high adsorption capacity of volatile organic compounds (VOCs) [59], acidic properties [60], high thermal stability and mechanical strength, catalytic properties [61], and dielectric properties [62,63]. The properties of PCH can be adjusted according to the targeted application. For example, the incorporation of different metals (e.g., Al, Zr) into the silica gallery can improve the thermal stability and increase the surface area and surface acidity. These properties make PCHs an efficient absorbent material for different pollutants (heavy metals, dyes). [64,65].

These versatile properties mean that PCHs can be used in a wide range of applications, such as in adsorbents [66,67,68], decontamination agents [69], and catalysts [70,71,72,73]. A new application of PCHs in the field of drug delivery systems was reported in our previous article [74].

In addition, we reported that the PCHs can be used as a nanofiller for NaAlg based films, inducing an increase of thermal stability and storage modulus [75].

Recently, hybrid beads based on PCHs and Na-Alg were synthesized and tested as adsorbents for volatile organic compounds [76].

In this study, some organic–inorganic hybrid hosts based on NaAlg and PCHs were synthesized and proposed as drug delivery systems for 5-Fluorouracil active substance. The performance of these materials as drug delivery systems may be influenced by the interaction between the components involved in the synthesis and the dispersion degree of PCHs within polymeric matrix, and the release of the drug is strongly influenced by the porous texture of PCHs and PCHs content.

## 2. Materials and Methods

### 2.1. Materials

**Figure 1:**
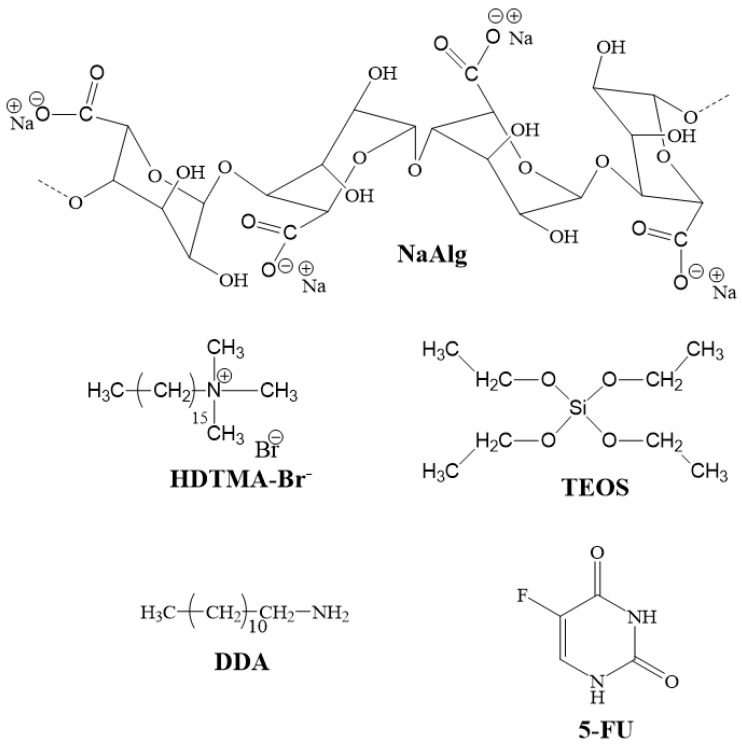
Chemical structure of raw materials.

Alginic acid sodium salt (NaAlg) from brown algae with medium viscosity was supplied from Sigma-Aldrich (St. Louis, MO, USA). Nanofil 116 (MMT-Na), a natural montmorillonite with a cationic exchange capacity (CEC) of 116 mEq/100 g clay was purchased from Southern Clay Products (Gonzales, TX, USA). Hexadecyltrimethylammonium bromide (HDTMA-Br), tetraethyl orthosilicate (TEOS), dodecylamine (DDA), and 5-fluorouracil (5-FU) were provided from Sigma–Aldrich and used as received Figure 1.

### 2.2. Synthesis of PCHs

The PCHs material was synthesized using the method described in our previous paper [54], which involves three main steps: (1) organophilization of montmorillonite raw material, (2) hydrolysis and polycondensation reaction of silica precursor in the presence of organically modified MMT, and (3) the final step focused on thermal treatment of PCHs precursors. 

In the first step, 10 g of MMT were subjected for the swelling process in 900 mL of demineralized water, and then 6 g of HDTMA was used as an organic agent to intercalate the swollen clay by cationic exchange reaction. The suspension was maintained for 5 h at 50 °C under mechanically stirring, and the final product was washed with water, isolated by centrifugation, and dried at room temperature.

The second step was focused on the synthesis of PCHs precursors using organically modified MMT that was treated with a precise amount of neutral amine (DDA) and silica precursor (TEOS) in the presence of water. The molar ratio used in the PCHs synthesis was 1:20:120 (modified MMT:DDA:TEOS).

The thermal treatment of PCHs precursors involves calcination at 650 °C for 6 h in air in order to remove the organic templates.

### 2.3. Synthesis of Hybrid Beads

**Figure 2:**
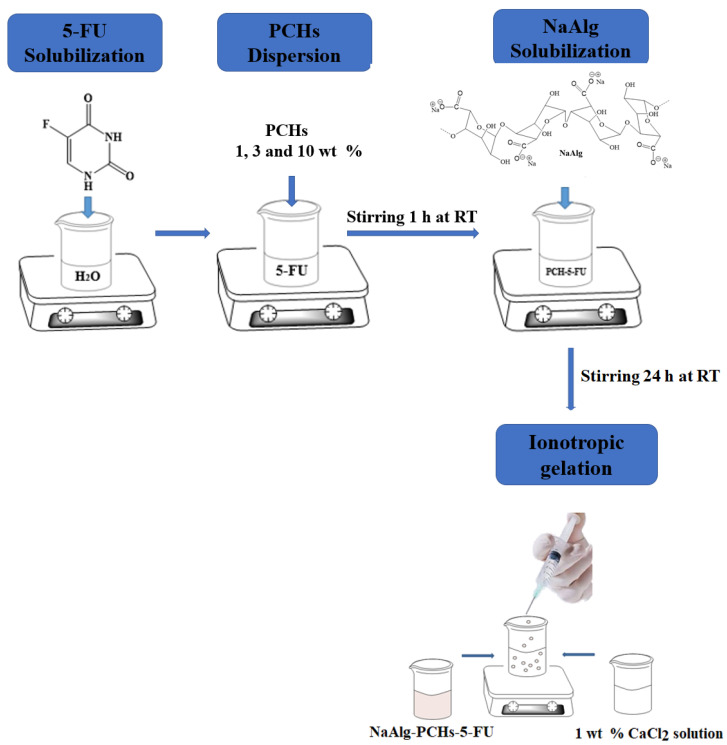
Synthesis steps of hybrid beads.

The preparation of hybrid beads based on NaAlg and PCHs was performed using the protocol shown in Figure 2. In the first step, 10 mg of 5-FU were dissolved in 10 mL of deionized water at room temperature (RT) under magnetic stirring. In the second step, different PCHs concentrations (1, 3, and 10 wt%) were dispersed in 5-FU solution under magnetic stirring for 1 h at RT. Into the obtained suspensions a certain amount of NaAlg was added to achieve 2% NaAlg solution, and then the hybrid systems (NaAlg-PCHs-5-FU) were stirred for 24 h in the absence of light at RT. In the final step, each suspension was dropped into calcium chloride solution (1 wt%) in order to obtain the hybrid beads by inotropic gelation. The hybrid beads were maintained in CaCl_2_ solution for 30 min and then were collected by filtration. After filtration, the supernatant was used to determine the encapsulation efficiency of 5-FU from hybrid beads. The amount of 5-FU entrapped in the NaAlg-PCHs system was determined using UV-Vis spectrophotometer (Cary 60, Agilent Technologies, Santa Clara, CA, USA) at λ = 265 nm. The beads were air dried for further characterization.

### 2.4. Characterization Techniques

Fourier Transform Infrared Spectrometry (FTIR) were recorded on an ATR Bruker VERTEX 70 spectrometer (Bruker, Billerica, MA, USA), using 32 scans with a resolution of 4 cm^−1^ in 4000–600 cm^−1^ wavenumbers range.

Thermogravimetric analysis (TGA) was done on Q 500 TA Instruments equipment (Bellingham, WA, USA), under inert atmosphere (nitrogen) and a heating rate of 10 °C/min.

The Quanta Inspect F50 scanning electron microscope (SEM) (FEI, Hillsboro, OR, USA), coupled with energy dispersive X-ray analysis (EDAX, USA), was used to evaluate the morphology of hybrid materials.

The drug encapsulation efficiency and in vitro drug release profiles of 5FU from different hybrid materials was investigated using a UV-Vis spectrophotometer (Cary 60) with a flow cell of 1 mm and a UV Dissolution software, coupled with a completely automated dissolution bath USP Apparatus 1 (708-DS Agilent, Agilent Technologies, Santa Clara, CA, USA) connected to an auto-controlled multichannel peristaltic pump (801 Agilent, Agilent Technologies, Santa Clara, CA, USA). The samples were put into a dialysis membrane bag with 4 mL buffer solution and then the samples were immersed in 200 mL dissolution medium (2 h in SGF and 22 h in SIF) and spin for 24 h with 70 rpm at 37 °C. At specific time intervals, the amount of released 5-FU was determined at λ = 265 nm. The hybrid hosts based on NaAlg and PCHs were analyzed in triplicate. The dissolution media were used without enzymes. 

## 3. Results

### 3.1. Characterization of Hybrid Beads

#### 3.1.1. FTIR Analysis

**Figure 3:**
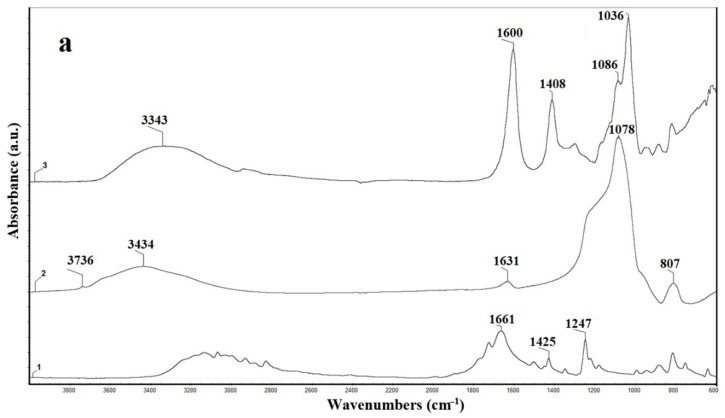

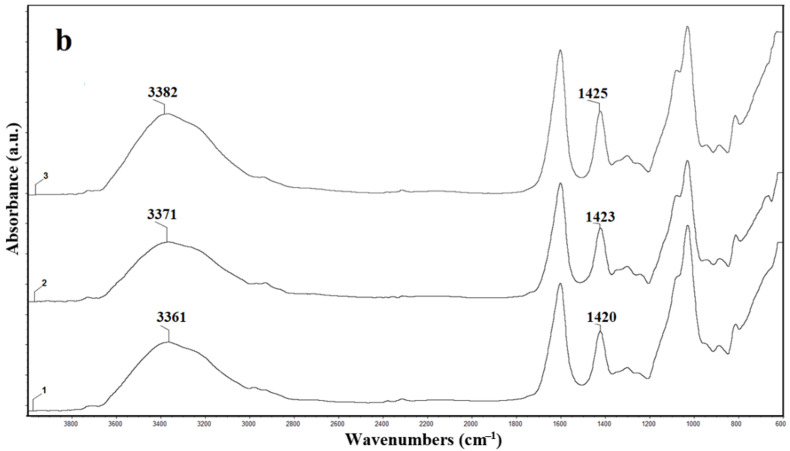
FTIR spectra of: (**a**): 1- 5-FU, 2-PCHs, 3- NaAlg; (**b**): 1- NaAlg- 5-FU-PCHs 1 wt%, 2- NaAlg- 5-FU-PCHs 3 wt%, 3- NaAlg- 5-FU-PCHs 10 wt%.

FTIR analysis was suitable to investigate the presence of clay (PCHs) and the antitumoral drug (5-FU) in the NaAlg beads, as well as to identify the possible interaction established between the components involved in the hybrid beads structures. The FTIR spectra of raw materials (NaAlg, PCHs, and 5-FU) and composite beads with various concentrations of PCHs (1, 3, and 10 wt%) are shown in Figure 3a,b. The FTIR spectra of 5-FU, NaAlg, and PCHs are similar to the ones reported in the literature [13,45,59,60,77,78]. In Table 1 are summarized the spectral assignments for raw materials.

As can be observed from Figure 3b, the presence of PCHs and the active substance into the polymeric matrix (NaAlg) was proven through the shifting to a higher value of the following peaks: (1) peak at 3343 cm^−1^ and (2) peak at 1408 cm^−1^ from NaAlg spectrum (Figure 3a)

The presence of the inorganic component (PCHs) into the polymeric matrix (NaAlg) induces a shifting of the peak from 3343 cm^−1^ to a higher value (3361/3371/3382 cm^−1^), which can be attributed to some physical interaction between NaAlg and PCHs (e.g., hydrogen bonding formation). Additionally, the FTIR data indicate that the shifting of this peak is significantly influenced by the PCHs concentration. The highest peak shifting was registered for hybrid materials with 10 wt% PCHs.

The presence of the active substance (5-FU) into the hybrid materials (NaAlg-PCHs) was highlighted by shifting the peak from 1408 cm^−1^ for NaAlg to higher values for hybrid materials (e.g., 1425 cm^−1^ for NaAlg-PCHs 10 wt%).

#### 3.1.2. TGA Tests

**Figure 4:**
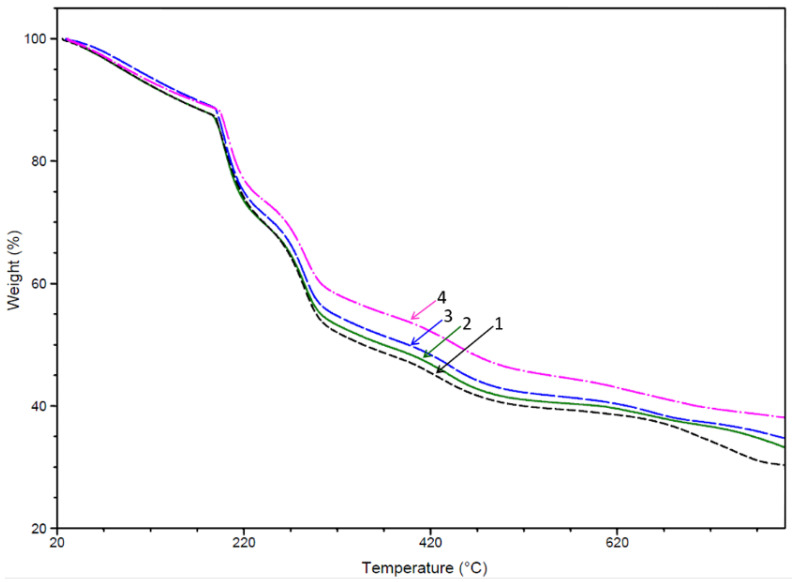
TGA curves of 1-NaAlg-5-FU, 2-NaAlg-5-FU-PCHs 1 wt%, 3-NaAlg-5-FU-PCHs 3 wt%, 4-NaAlg-5-FU-PCHs 10 wt%.

TGA tests were performed to demonstrate the presence of PCHs in drug loaded hybrid beads. PCHs material exhibits a different thermal degradation profile [74], characterized by a higher thermal stability, and therefore the hybrid materials based on the NaAlg matrix and PCHs exhibited different thermal properties depending on PCHs content. As shown in Figure 4 and Table 2, the presence of PCHs was confirmed by the increase of thermal stability of the drug loaded hybrid beads.

All the hybrid materials exhibit a similar TG profile to the 5-FU loaded NaAlg beads, but the presence of PCHs induces a barrier effect, and therefore a slight increase of degradation temperatures (T_d15 %_ and T_d40 %_) was recorded. This effect was mainly observed for hybrids beads with high content of PCHs (10 wt%).

A similar trend was also observed by other authors for various materials based on NaAlg and different nanostructured agents such as graphene oxide (GO), Na-montmorillonite, and layered double hydroxide (LDH) [34,79,80].

#### 3.1.3. SEM Characterization

**Figure 5:**
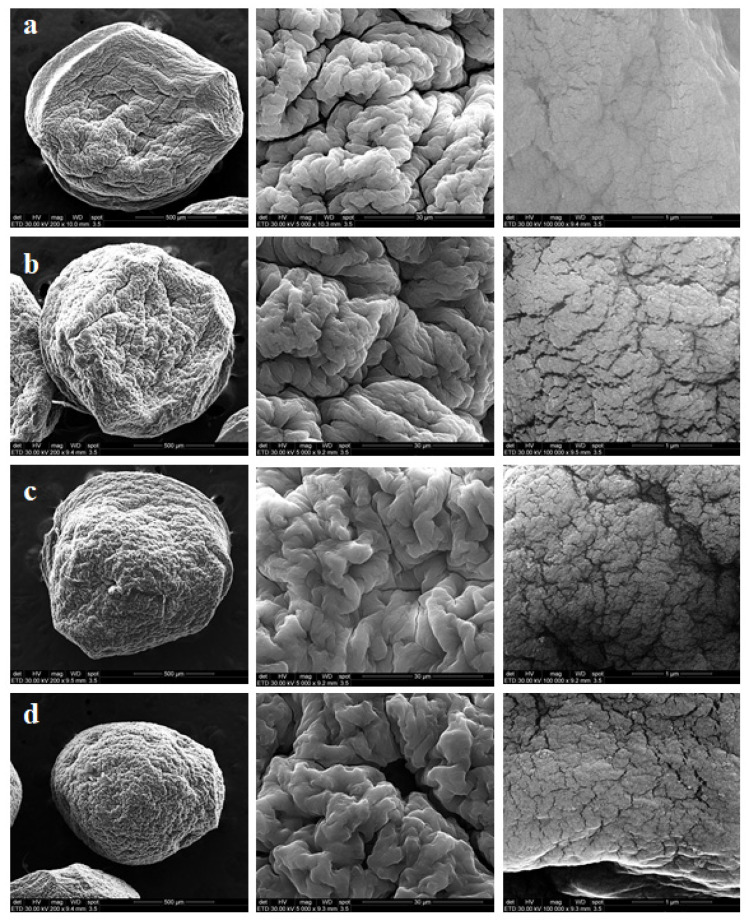
SEM images of the beads with different PCHs concentrations: (**a**) neat NaAlg, (**b**) 1 wt%, (**c**) 3 wt%, and (**d**) 10 wt%.

SEM analysis was employedto investigate the surface morphology of hybrid beads (Figure 5).

The SEM micrographs reveal that dried hybrid beads based on NaAlg and PCHs were characterized by a rough surface with visible wrinkles and a spherical shape after drying. The SEM images of NaAlg beads indicate a smooth surface that suggests the presence of a uniform structure. The uniform dispersion of PCHs into the core structure of hybrid beads was highlighted by the SEM results. Even at high PCHs concentrations (10 wt%), the clay did not exhibit a tendency to form clusters. 

Similar results were reported for the hybrid systems alginate/bentonite/imidacloprid [81] and alginate/montmorillonite/curcumin [82].

**Figure 6:**
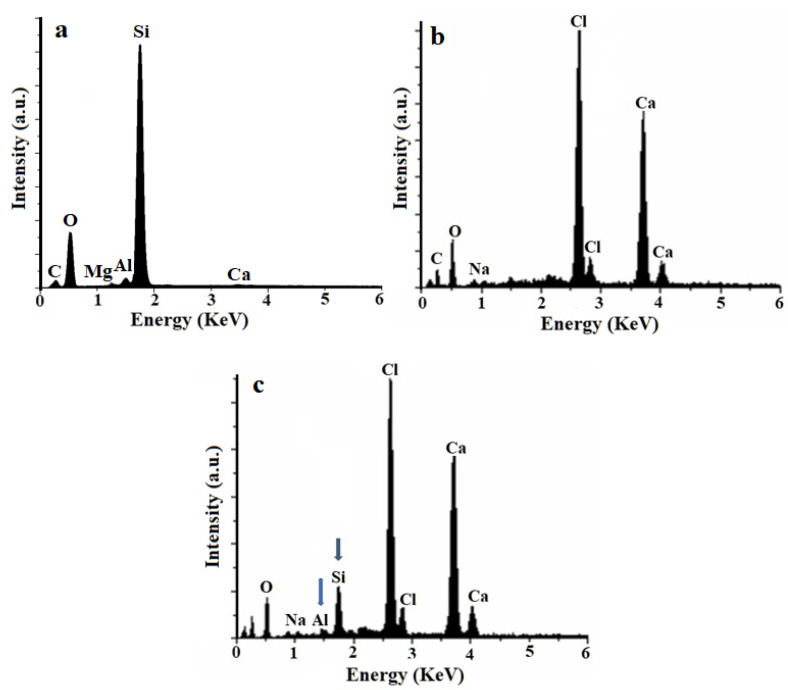
EDAX spectra for: (**a**) PCHs, (**b**) Neat NaAlg beads, and (**c**) hybrid materials NaAlg-PCHs 10 wt%.

Energy dispersive X-ray spectroscopy (EDAX) is a useful method to confirm the presence of PCHs in the NaAlg matrix. The EDAX spectrum of PCHs confirms the presence of characteristic signals such as Si and O, which are the major elements of the clay (Figure 6a).The neat NaAlg hydrogel exhibits the characteristic peaks of a crosslinked network (Ca, Cl) (Figure 6b).

The EDAX spectra of hybrid beads (Figure 6c) based on NaAlg and PCHs show the presence of peaks assigned for Si and Al atoms, which confirmed the presence of PCHs in the polymer.

#### 3.1.4. Determination of the Encapsulation Efficiency and Drug Release Profile

Encapsulation efficiency of 5-FU and the amount of 5-FU released from the hybrid beads systems based on NaAlg and PCHs were determined using the UV-Vis method.

The 5-FU encapsulation efficiency was calculated using Equation (1).

(1) $$EE\;\left(\%\right)=\frac{W_{\mathrm{total}\;5-\mathrm{FU}}-W_{\mathrm{free}\;5-\mathrm{FU}}}{W_{\mathrm{total}\;5-\mathrm{FU}}}\times 100$$

where *W*_total 5-FU_ is the initial 5-FU amount and *W*_free 5-FU_ is the unloaded 5-FU amount.

The UV-Vis results confirmed the PCHs influence in the NaAlg beads. The PCHs concentration influences the encapsulation efficiency (*EE%*) and release profile of 5-FU from hybrid beads.

As shown in Table 3, the values of drug *EE* registered for hybrid beads (NaAlg-PCHs) that contain different concentrations of PCHs (1, 3, 10 wt%) are significantly changed.

The presence of PCHs induces a noticeable increase of *EE* by comparing with the NaAlg-5-FU. The lowest drug *EE* value was recorded for the neat NaAlg beads (60%), and the hybrid beads (NaAlg-5-FU-PCHs-1, 3, 10 wt%) exhibit a higher 5-FU encapsulation efficiency (70%). This fact can be attributed to the properties of PCHs (textural properties, large surface area, high porosity, and noticeable adsorbent capacity), which highlights the importance of PCHs for the increase of drug encapsulation efficiency.

**Figure 7:**
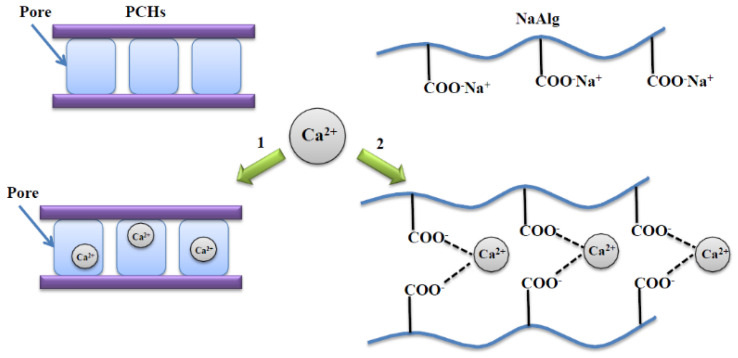
Possible interaction of NaAlg and PCHs with divalent cation (Ca^2+^) in ionotropic gelation process.

In these hybrid beads, two types of interactions play a crucial role on the drug release profile. These interactions are schematically described in Figure 7 and include: (1) polymer-divalent cation interaction and (2) clay-divalent cation interaction.

PCHs can adsorb Ca^2+^ ions into the porous structure, and therefore a significant decrease of CaCl_2_ solution concentration involved in the ionotropic gelation process occurs. The high adsorption capacity of PCHs has also been reported in the literature [69].

**Figure 8:**
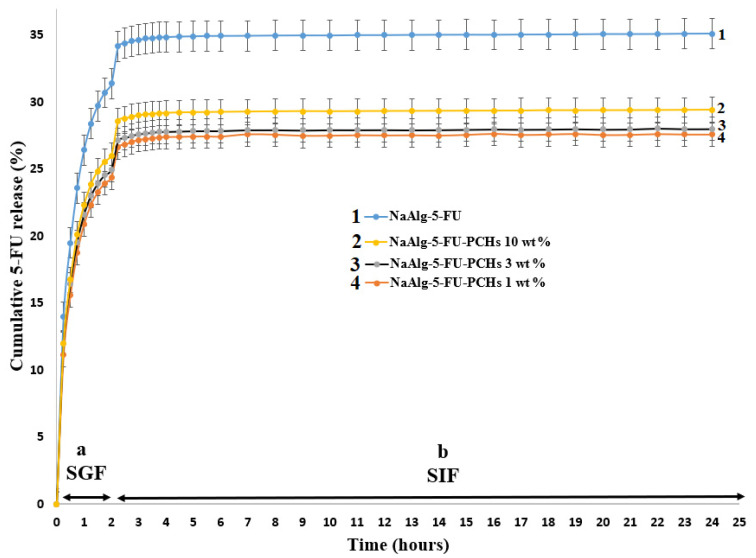
Drug release profiles in SGF (a) and SIF (b) for: 1-NaAlg-5-FU, 2-NaAlg-5-FU-PCHs 10 wt%, 3-NaAlg-5-FU-PCHs 3 wt%, 4-NaAlg-5-FU-PCHs 1 wt%.

The release profiles of 5-FU from hybrid materials in SIF and SGF are presented in Figure 8.

As can be observed in Figure 8, the drug release profile can be influenced with the increase of PCHs concentration. In both SGF and SIF, the highest amount of drug release is registered for NaAlg-5-FU (35%), followed by NaAlg-5-FU-PCHs 10 wt% (29%), NaAlg-5-FU-PCHs 3 wt% (28%), and then NaAlg-5-FU-PCHs 1 wt% (27%). The drug release concentration decreases with the decrease of PCHs amount. This may be caused by the possibility that PCHs adsorbs a part of the Ca^2+^ ions, and therefore the final structure of the beads was affected. In addition, the presence of a high PCHs content (10 wt%) into NaAlg beads induces a structure with a high porosity that allows an easier diffusion of drug molecules through the polymer matrix. In the SGF (pH = 1.2), the hybrid beads present a progressive release of 5-FU, and in SIF a linear release of 5-FU was recorded. This may be attributed to the sensitivity of NaAlg to pH changes [83].

These results suggest that the presence of PCHs into NaAlg induced a decrease of 5-FU release from hybrid beads and also induce a decrease of the NaAlg hydrogel burst release issue. This phenomenon can be attributed to the barrier effect of PCHs. Similar results were reported for sodium alginate/layered double hydroxides/diclofenac [84].

## 4. Conclusions

New hybrid materials with potential applications in cancer therapy were successfully prepared. These materials, based on NaAlg and PCHs, can be considered as possible hybrid host candidates for 5-FU encapsulation.

The presence of PCH inorganic nanomaterial, characterized by a high specific surface area, high porosity, and good adsorption capacity within NaAlg beads strongly influenced the 5-FU encapsulation efficiency, and the drug release profile can be adjusted by using different PCHs concentration. The burst release can be significantly diminished by adding lower PCHs concentrations (1 wt%). The hybrids that include higher PCHs content (10 wt%) are characterized by a faster drug release rate.

All the results proved that the hybrid materials based on NaAlg and PCHs exhibit superior properties compared to the neat classical NaAlg beads for the encapsulation and release of 5-FU.

## Figures and Tables

**Table 1:** Assignment of the characteristic peak of raw materials.

NaAlg		PCH		5-FU	
Wavenumber (cm^−1^)	Type of vibrations	Wavenumber (cm^−1^)	Type of vibrations	Wavenumber (cm^−1^)	Type of vibrations)
3343	Symmetric stretching vibration of hydroxyl group (OH)	3736	Stretching vibration of the OH group from Si–OH	1661	Stretching vibration C=C Stretching vibration of carbonyl group (C=O)
1600	Symmetric stretching vibration of carboxylate group (COO^−^)	3434	Stretching vibration of the OH group of water molecules adsorbed on PCH	1425	Bending vibration of N–H
1408	Asymmetric stretching vibration of carboxylate group (COO^−^)	1631	Bending vibration of adsorbed water molecules	1247	Stretching vibration of the aromatic ring
1086 1036	Stretching vibration of C–O–C	1078	Stretching vibrations of three dimensional silica network	-	-
-	-	807	Symmetric stretching vibrations of Si–O–Si or Si–O–Al	-	-

**Table 2:** Thermal properties of hybrid beads.

Sample	T_d15%_ (°C) *	T_d 40%_ (°C) **
NaAlg-5-FU	194	284
NaAlg-5-FU-PCHs 1 wt%	194	285
NaAlg-5-FU-PCHs 3 wt%	196	287
NaAlg-5-FU-PCHs 10 wt%	201	303

* T_d15%_-the temperature at which the weight loss is 15 % ** T_d40%_-the temperature at which the weight loss is 40 %.

**Table 3:** 5-FU encapsulation efficiency (*EE*, %) of neat NaAlg beads and hybrid beads.

Sample	*EE*, %
NaAlg-5-FU	60
NaAlg-5-FU-PCHs 1 wt%	70
NaAlg-5-FU-PCHs 3 wt%	70
NaAlg-5-FU-PCHs 10 wt%	70

## Data Availability

Not applicable.
